# Physical activity and associated levels of disability and quality of life in people with multiple sclerosis: a large international survey

**DOI:** 10.1186/1471-2377-14-143

**Published:** 2014-07-12

**Authors:** Claudia H Marck, Emily J Hadgkiss, Tracey J Weiland, Dania M van der Meer, Naresh G Pereira, George A Jelinek

**Affiliations:** 1 Emergency Practice Innovation Centre, St Vincent’s Hospital Melbourne, PO Box 2900, Fitzroy 3065, VIC, Australia; 2 Department of Medicine, The University of Melbourne (St Vincent’s Hospital), Fitzroy, Victoria, Australia; 3 Faculty of Medicine, Notre Dame University, Fremantle, Western Australia, Australia; 4 Department of Epidemiology and Preventive Medicine, Monash University, Melbourne, Victoria, Australia

**Keywords:** Multiple sclerosis, Exercise, Physical activity, Quality of life, Disability, Rehabilitation

## Abstract

**Background:**

Multiple Sclerosis (MS) is a common neurodegenerative disease, which often has a devastating effect on physical and emotional wellbeing of people with MS (PwMS). Several studies have shown positive effects of physical activity (PA) on disability, health related quality of life (HRQOL), and other outcomes. However, many studies include only people with mild disability making it difficult to generalize findings to those with moderate or severe disability. This study investigated the associations between PA and HRQOL, relapse rate (RR), disability, and demographic variables in PwMS with varying disability.

**Methods:**

Through online platforms this large international survey recruited 2232 participants with MS who completed items regarding PA, MS and other health characteristics.

**Results:**

PwMS who were younger (p < .001), male (p = 0.006), and with lower body mass index (BMI) (p < .001) undertook more PA, which was associated with decreased disability (p < 0.001) and increased HRQOL measures (all p < 0.001). For the subsample of people with relapsing-remitting MS, PA was associated with a decreased RR (p = 0.009). Regression analyses showed that increased PA predicted clinically significant improvements in HRQOL while controlling for level of disability, age and gender. More specifically, increasing from low to moderate and to high PA increased estimated mean physical health composite from 47.7 to 56.0 to 59.9 respectively (25.6% change), mental health composite from 60.6 to 67.0 to 68.8 (13.5% change), energy subscale from 35.9 to 44.5 to 49.8 (38.7% change), social function subscale from 57.8 to 66.1 to 68.4 (18.3% change), and overall QOL subscale from 58.5 to 64.5 to 67.7 (15.7% change).

**Conclusions:**

For PwMS, regardless of disability level, increased PA is related to better HRQOL in terms of energy, social functioning, mental and physical health. These are important findings that should be taken into consideration by clinicians treating PwMS.

## Background

Multiple sclerosis (MS) is an increasingly common neurodegenerative disorder affecting approximately 1 in 1000 people in Australia [1] and the USA [2]. Progressive loss of function common in people with MS (PwMS) often has a devastating effect on physical and emotional wellbeing. In the general population physical activity (PA), or exercise, has known wide-ranging benefits for physical and emotional wellbeing [3,4] and is generally thought to also have benefits for PwMS [5,6]. However, increasing disability is linked to decreasing levels of PA [7,8], as hallmark MS symptoms such as pain, weakness, and problems with balance may limit a person’s ability to participate in PA. For many PwMS fatigue is a major debilitating symptom and they may feel they need to conserve energy. Conversely, a lack of PA, or inactivity, may contribute to an increased risk of comorbidities or obesity which are known to negatively affect disease progression in MS [9] and may also lead to deconditioning and muscle weakness [10]. Furthermore, PwMS generally have lower levels of quality of life (QOL) compared to the general population [11], and although the evidence is inconclusive [12,13], several studies have reported beneficial effects of PA on QOL in PwMS [14-16].

Many trials investigating the effects of PA on MS have included only a small number of people, usually with mild to moderate disability, not experiencing any exacerbations, and study outcome measures were found to be inconsistent. Furthermore, some studies fail to report important sample characteristics or correct for level of disability. Thus, while there seems to be an overall positive effect of PA, the evidence is inconclusive; and more research is needed to be able to draw strong conclusions [5,17-19].

### Aim

In the current study, which is part of a large international survey of people with MS and the first baseline data collected as part of a longitudinal dataset, we investigate the associations between self-initiated PA or exercise, and HRQOL, relapse rate, disease activity and disability, as well as demographic variables.

## Methods

The methods of the larger Health Outcomes and Lifestyle Interventions in a Sample of People with Multiple Sclerosis (HOLISM) study have been described in detail elsewhere [20] but will be summarized here. The study was approved by the Human Research Ethics Committee of St Vincent’s Hospital Melbourne (LRR 055/12).

### Participants and data collection

Participants were recruited through online MS societies and platforms including websites, Facebook pages, twitter, blogs and forums where the survey was advertised. All media were specifically for people with MS, some actively promoted healthy lifestyle and most were based in English speaking countries including the US, Australia, New Zealand and the UK. The survey was in English and included a participant information and consent page which required a response before entering the survey. Participants were aged over 18 years and reported a physician confirmed diagnosis of MS. Contact details were recorded for future follow-ups as this is part of a longitudinal study.

### Tools

The online survey comprised several validated questionnaires as well as researcher devised or amended items, and took approximately 40 minutes in total to complete with the option to come back to it at a later time if it was not completed at once. All data were self-reported.

#### ***Independent variables***

Several multiple choice items assessed demographical variables age, gender, height, and weight.

The International Physical Activity Questionnaire (IPAQ) short form is a 7-day recall self-administered questionnaire which measures PA as well as time spent sitting over the last 7 days [21]. Scores from the abbreviated IPAQ correlate moderately with measures from a pedometer (r = 0.32) and an accelerometer (r = 0.36) in individuals with MS [22] and can be used to successfully estimate maximal oxygen uptake (VO(2)max) [23]. To measure PA, items assessed walking, moderate and vigorous intensity activities with frequency measured in days per week and duration measured in hours and minutes per day. Examples of moderate exercise (e.g., doubles tennis or regular cycling) and vigorous exercise (e.g., heavy lifting, aerobics or fast cycling) were provided. These measures were then combined to form a total activity score, MET (multiples of the resting metabolic rate)-minutes per week, and a categorical variable. Instructions for data cleaning and scoring were followed as per the IPAQ scoring instructions [24]. All values under 10 minutes per day were omitted and values over 240 minutes per day per type of activity were truncated to 240. “Minutes per week” was calculated by combining “hours” and “minutes” for duration of activity, and multiplying this by “days per week”. If “times per week” for one of the categories was left blank, it was assumed that the “hours per day” and “minutes per day” were a weekly total. If “times per week” for one of the categories was completed but both “hours” and “minutes” was left blank, this category returned a weekly total of zero. A MET-minutes per week score was obtained by multiplying walking minutes per week by 3.3, moderate activity by 4 and vigorous activity by 8 and then finally combining these scores together.

The categorical variable had the following categories (adapted from IPAQ scoring protocol [24]): 

High active: Vigorous-intensity activity on at least 3 days and accumulating a minimum of 1500 MET-min/week; or 7 or more days of any combination of walking, moderate-intensity or vigorous intensity activities achieving a minimum of 3000 MET-min/week

Moderately active: 3 or more days of vigorous activity of at least 20 minutes per day; or 5 or more days of moderate-intensity activity or walking of at least 30 minutes per day; or 5 or more days of any combination of walking, moderate-intensity or vigorous intensity activities achieving a minimum of 600 MET-min/week.

Low active: No activity is reported or some activity is reported but not enough to meet moderate or high categories.

The IPAQ was slightly amended to suit the online survey, and instructions were as follows: “Please answer the following questions about physical activity even if you do not consider yourself to be an active person or your MS significantly limits your ability to exercise.”

Disability was measured by the patient-determined disease steps (PDDS) [25-27]. The PDDS is a self-administered surrogate tool to the commonly used clinician-administered expanded disability status scale (EDSS). It is scored from 0 (normal) to 8 (bed bound) and has been collapsed into three categories for the purpose of data analysis in this paper: scores of 0, 1 and 2 were categorized as low disability; scores of 3, 4 and 5 were categorized as moderate disability; and scores of 6, 7 and 8 were categorized as high disability.

#### ***Dependent variables***

Physician-diagnosed relapse rates over the last 12 months and 5 years were obtained for those with relapsing remitting MS only.

Body Mass Index (BMI) was obtained by multiplying weight (in kilograms) by height^2^ (in centimeters). BMI was recoded into categories according to the World Health Organization (WHO) so that those with BMI below 18.5 were classed as underweight, those with BMI of 18.5 and up to 25 normal, BMI of 25 and up to 30 overweight and BMI of 30 and over obese [28].

Disease activity (for those with relapsing-remitting MS only) was categorized as increasing, decreasing, or stable depending on whether the relapse rate in the preceding 12 months was higher, lower, or the same, respectively, as the average annualized relapse rate over the disease duration, capped to the last five years.

HRQOL was measured by the widely used and validated Multiple Sclerosis Quality Of Life-54 (MSQOL-54) [29]. The MSQOL-54 consists of 52 items, including the 36-item Short Form health survey (SF-36), which generates two composite scores: the physical and mental health composites and 12 subscales, and two additional items. The MSQOL-54 was scored according to the scoring instructions, but only the two composite scores and those scales relevant to PA were used for analysis: physical health composite (PHC), mental health composite (MHC), energy subscale (ES), social function subscale (SFS), and overall quality of life subscale (OQOLS).

### Data analysis

Data were analyzed using SPSS version 22.0 (IBM Corporation). Continuous data were reported using mean (95% CI) or median (IQR) and categorical data using number (N) and percentage (%). Varying denominators are due to varying item completion. Analyses were only performed for those reporting a physician confirmed diagnosis of MS. The IPAQ produced a continuous variable of “MET minutes”, which was very positively skewed and could not be transformed, and a categorical variable. We used the categorical variable for analysis.

Pearson’s Chi Square was used to compare categorical variables, or Fisher’s exact test for two by two contingency tables with standardized adjusted residuals indicating over- or underrepresentation of groups. Analysis of variance was used to compare continuous variables across three or more groups with planned comparisons for post hoc testing. A Welch correction was carried out if the homogeneity of variances assumption was violated; this was the case for most variables except age, the MSQOL-54 ES and PHC. Alpha was set at 0.05 and two tailed tests of significance were used in all instances. These analyses for demographic variables were repeated for each of the three disability groups separately to control for disability level.

Regression analyses were carried out to identify independent predictors of outcomes. No regression analysis was carried out to predict disability as inverse causality seemed probable. Multiple regression (enter method) was used to identify predictors of PHC, MHC, SFS, OQOLS, and ES. It was ensured that data satisfied the assumptions of normality, linearity, and homoscedasticity by plotting the studentized residuals against the unstandardized predicted values of the dependent variable and assessing the spread. The MHC score had a skewed distribution of standardized residuals and a sensitivity analysis was undertaken. Independence of residuals was assessed by checking the Durbin Watson statistic (values between 1 and 3 were accepted). Variance inflation factor <5 was used as the criterion for absence of multicollinearity. Only correlations of <.70 between predictor variables were accepted as inspected from the correlation matrices. Data were checked for standardized residual values outside the range of -3.0 to 3.0 and outliers were inspected. There were no influential cases with a Cook value over 1. As data for 12 month relapse rate among those with relapsing-remitting MS were over-dispersed, negative binomial logistic regression was used after verifying that the assumptions were met in regards to multicollinearity (cut-off was >0.70). For disease activity (decreasing/stable versus increasing), binary logistic regression (enter method) was planned but not undertaken as the bivariate analysis showed no significant results. Parameter estimates and estimated marginal means (EMM) were reported as well as percentage change by dividing the difference between EMM of low level of PA and high level of PA, by the EMM of low level of PA. All percentages reported were adjusted for missing data or item non-completion.

## Results

### Demographic variables

Demographics of the whole sample (N = 2469) have been described in detail elsewhere but to summarize, participants were overall highly educated, and lived in 57 mostly English speaking countries [20]. Here we describe demographic and clinical characteristics of those 2,232 participants with a self-reported physician confirmed diagnosis of MS who completed sufficient data items to have an IPAQ score computed (Table 1).

**Table 1:** Demographic variables and clinical characteristics

**Continuous demographic variables**	**Mean**	**95% CI**	**Median**	**IQR**	**N**
Age	45.7	45.3-46.2	46.0	38.0-53.0	2172
Weight (kg)	72.0	71.3-72.8	68.0	59.0-80.9	2217
Height (cm)	167.6	167.2-168.0	167.0	162.0-173.0	2218
BMI	25.7	25.4-26.0	24.0	21.3-28.2	2208
Years since diagnosis	8.7	8.4-9.0	6.0	3.0-12.0	2224
12 month relapse rate*	0.71	0.65-0.77	0.0	0.0-1.0	1330
MSQOL Energy subscale	43.6	42.7-44.5	44.0	24.0-60.0	2225
MSQOL Social function subscale	69.7	68.7-70.7	75.0	50.0-91.7	2159
MSQOL Overall QOL subscale	67.1	66.3-67.9	68.4	55.0-81.7	2181
MSQOL Physical Health Composite	59.3	58.3-60.3	59.2	42.6-77.8	1877
MSQOL Mental Health Composite	67.0	66.1-67.9	72.6	52.0-84.1	2136
**Categorical variables**	**N**	**%**			**N**
*Gender*					2208
Female	1819	82.4			
Male	389	17.6			
*Type of MS*					2226
Relapsing-remitting	1362	61.2			
Secondary progressive	258	11.6			
Primary progressive	163	7.3			
Benign	94	4.2			
Progressive relapsing	46	2.1			
Unsure/other	303	13.6			
Disease Activity*					1263
Decreasing	537	42.5			
Same	343	27.2			
Increasing	383	30.3			
*Body Mass Index*					2208
Underweight	92	4.2			
Normal	1182	53.5			
Overweight	510	23.1			
Obese	424	19.2			
*Disability*					2223
Low	1219	54.8			
Moderate	769	34.6			
High	235	10.6			

*Includes people with relapsing-remitting MS only.

Frequencies, medians as well as means of the METS minutes are reported here to be able to compare it to those studies which have previously reported means (Table 2).

**Table 2:** Physical activity measures

**Continuous IPAQ measures**	**Median**	**Interquartile range**	**Mean**	**Std. deviation**	**Range**	**N**
Total METS minutes	1053.0	297.0-2719.0	1901.4	2336.8	.0-18558.0	2232
Sitting minutes per week	3360.0	2100.0-4200.0	3299.2	1602.9	.0-8400.0	2125
**IPAQ categories**	**%**					**N**
Low active	41.3					922
Moderate active	31.7					707
High active	27.0					603

### Physical activity and demographics

#### ***Disability***

Level of disability was strongly related to the level of PA, those with a higher level of disability had a lower level of PA and vice-versa (p < 0.001) (Table 3).

**Table 3:** Association between levels of physical activity and disability

**Level of disability**	**Physical activity level**		
	**Low**	**Moderate**	**High**
**Low**	323	445	451
	26.5%^	36.5%*	37.0%*
**Moderate**	401	234	134
	52.1%*	30.4%	17.4%^
**High**	193	26	16
	82.1%*	11.1%^	6.8%^
Total	917	705	601
	41.3%	31.7%	27.0%

^indicates underrepresentation *indicates overrepresentation.

#### ***Gender***

PA was significantly related to gender (p = 0.006) with males more often highly active compared to females (33.2% vs 25.6%) and less often low active compared to females (36.2% vs 42.6%). Further analysis showed that gender was only related to levels of PA for those in the low disability group (p = 0.006) with males more often highly active compared to females (46.5% vs 35.1%) and less often low active compared to females (19.7% vs 27.7%).There was a trend for those in the moderate disability group (p = 0.053) with males less often low active compared to females (44.7% vs 54.9%). For those in the high disability group there was no significant association between gender and levels of PA.

#### ***Age***

PA was related to age (p < 0.001), and planned comparisons (post-hoc testing) showed that those with low levels of PA were older (47.3 years, CI 46.7-48.0) compared to those with moderate (45.1, CI 44.3-45.9 years) and high (43.9 years, CI 43.1-44.8) levels of PA (p < 0.001 for both) and the difference between moderate and high levels of PA was also significant (p = 0.036). Further analysis showed that age was not significantly related to PA within any of the 3 disability levels.

#### ***Body Mass Index***

PA was significantly related to BMI (p < 0.001) and planned comparisons showed that those with low levels of PA had significantly higher BMI (26.8, CI 26.3-27.3) compared to those with moderate (25.1, CI 24.6-25.5) and high (24.8, CI 24.3-25.2) levels of PA (p < 0.001 for both comparisons). No significant difference in BMI between moderate and high levels of activity was found. Additional analyses within level of disability showed that for those with low disability, BMI was significantly related to PA (p < 0.001), with planned comparisons showing significant differences in BMI between low (26.7, CI 26.0-27.5) and moderate (24.9, CI 24.4-25.4), and low and high (24.3, CI 23.9-24.8) levels of PA (both P < 0.001). For those with moderate disability, BMI was significantly related to PA (p = 0.006), with planned comparisons showing significant differences between the BMI for those with low (27.2, CI 26.4-27.9) and moderate (25.4, CI 24.6-26.2) levels of PA (P = 0.002). For those in the high disability category, BMI was not significantly related to PA.

### Physical activity and outcome variables

#### ***Relapse rate***

For those with relapsing-remitting MS only, the level of PA was related to the 12 month relapse rate (p = 0.009). Planned comparisons showed that those with low levels of PA had more annual relapses (0.8, CI 0.7-1.0) compared to those with moderate (0.7, CI 0.6-0.8, p = 0.024) and those with high levels of PA (0.6, CI 0.5-0.7, p = 0.004) with no significant difference between moderate and high levels of PA. Negative binomial regression modelling showed that in a model with age, gender, disability and PA, PA was not a significant predictor of 12 month relapse rate (results not shown).

#### ***Disease activity***

For those with relapsing-remitting MS only, PA was found not to be related to disease activity. Therefore, no regression modelling was undertaken.

#### ***Quality of life***

**Figure 1:**
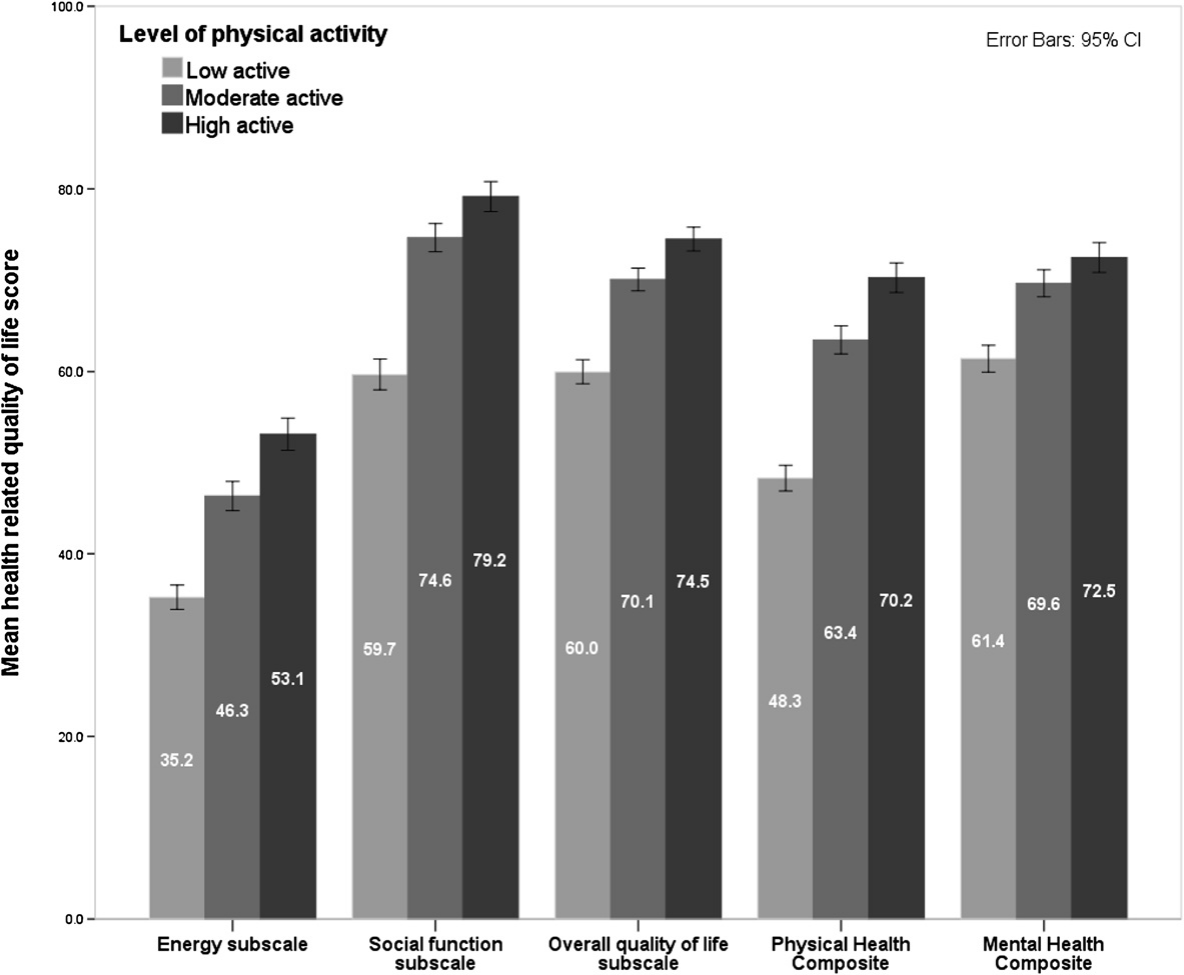
Associations between health related quality of life measures and physical activity.

All subscales and composite scores of the MSQOL-54 were significantly related to PA and post-hoc tests showed that all subscales and composite scores were significantly different between low vs. moderate, low vs. high, and moderate vs. high levels of physical activity (all <.001 except between moderate and high activity on the mental health composite (p =.012)) (Figure 1).

Regression analyses showed that the model including age, gender, disability and PA significantly predicted PHC, MHC, ES, SFS and OQOLS scores, explaining between 10.6 and 43.5% of the variance (Table 4). Higher disability level was significantly associated with lower HRQOL scores, controlling for PA, age and gender. MHC, ES, SFS, and OQOLS scores increased with age and while male gender predicted a higher score on the PHC and ES it predicted a lower score on the OQOLS (Table 4).

**Table 4:** Significant predictors and covariates for components of HRQOL

**Outcome variable**	**Predicting variable**	**Adjusted R**^**2**^	**Beta**	**SE**	**p**
**Physical health composite**	**Intercept**	**.435 P < .001**	**61.209**	**1.832**	**<.001**
	Age		.037	.040	.355
	**Male gender**		**2.393**	**.974**	**.014**
	**Moderate level of physical activity**		**8.331**	**.944**	**<.001**
	**High level of physical activity**		**12.263**	**.998**	**<.001**
	**Moderate level of disability**		**-22.172**	**.898**	**<.001**
	**High level of disability**		**-26.950**	**1.451**	**<.001**
**Mental health composite**	**Intercept**	**.106 P < .001**	**51.818**	**2.125**	**<.001**
	**Age**		**.341**	**.045**	**<.001**
	Male gender		.068	1.168	.954
	**Moderate level of physical activity**		**6.369**	**1.097**	**<.001**
	**High level of physical activity**		**8.175**	**1.176**	**<.001**
	**Moderate level of disability**		**-10.699**	**1.047**	**<.001**
	**High level of disability**		**-9.457**	**1.653**	**<.001**
**Energy subscale**	**Intercept**	**.167 P < .001**	**31.844**	**2.149**	**<.001**
	**Age**		**.224**	**.046**	**<.001**
	**Male gender**		**2.783**	**1.185**	**.019**
	**Moderate level of physical activity**		**8.590**	**1.114**	**<.001**
	**High level of physical activity**		**13.927**	**1.189**	**<.001**
	**Moderate level of disability**		**-12.872**	**1.063**	**<.001**
	**High level of disability**		**-9.838**	**1.672**	**<.001**
**Social function subscale**	**Intercept**	**.292 P < .001**	**67.104**	**2.135**	**<.001**
	**Age**		**.141**	**.045**	**.002**
	Male gender		.784	1.179	.506
	**Moderate level of physical activity**		**8.304**	**1.109**	**<.001**
	**High level of physical activity**		**10.544**	**1.184**	**<.001**
	**Moderate level of disability**		**-18.749**	**.594**	**<.001**
	**High level of disability**		**-29.595**	**1.058**	**<.001**
**Overall quality of life subscale**	**Intercept**	**.196 P < .001**	**63.904**	**1.770**	**<.001**
	**Age**		**.109**	**.038**	**.004**
	**Male gender**		**-2.432**	**.977**	**.013**
	**Moderate level of physical activity**		**6.086**	**.917**	**<.001**
	**High level of physical activity**		**9.247**	**.976**	**<.001**
	**Moderate level of disability**		**-11.700**	**.873**	**<.001**
	**High level of disability**		**-15.867**	**1.372**	**<.001**

Female gender, low level of activity and low level of disability were used as reference categories in regression analyses.

Bold indicates significant finding at <0.05 level.

PA significantly increased all HRQOL measures, controlling for age, gender and disability level (Table 5).

**Table 5:** Estimated marginal means for health related quality of life measures by gender, disability and physical activity

**Gender**	**Level of disability**	**Level of physical activity**	**Physical health composite**	**Mental health composite**	**Energy subscale**	**Social function subscale**	**Overall QOL subscale**
			**EMM**	**EMM**	**EMM**	**EMM**	**EMM**
			**SE**^**a**^	**SE**^**b**^	**SE**^**c**^	**SE**^**d**^	**SE**^**e**^
Male	High	High	50.6	66.1	48.9	55.3	59.8
			1.7	2.0	2.0	2.0	1.6
		Moderate	46.6	64.3	43.6	53.1	56.6
			1.7	1.9	2.0	2.0	1.6
		Low	38.3	57.9	35.0	44.7	50.6
			1.5	1.7	1.7	1.7	1.4
	Moderate	High	55.3	64.9	45.9	66.1	64.0
			1.2	1.5	1.5	1.5	1.2
		Moderate	51.4	63.1	40.6	63.9	60.8
			1.2	1.4	1.4	1.4	1.2
		Low	43.1	56.7	32.0	55.6	54.7
			1.1	1.3	1.3	1.3	1.1
	Low	High	77.5	75.6	58.8	84.9	75.7
			1.1	1.3	1.3	1.3	1.1
		Moderate	73.6	73.8	53.4	82.6	72.5
			1.1	1.3	1.3	1.3	1.1
		Low	65.3	67.4	44.9	74.3	66.4
			1.2	1.4	1.4	1.4	1.2
Female	High	High	48.2	66.0	46.2	54.5	62.2
			1.5	1.8	1.8	1.8	1.5
		Moderate	44.2	64.2	40.8	52.3	59.1
			1.5	1.7	1.7	1.7	1.4
		Low	35.9	57.9	32.2	44.0	53.0
			1.3	1.4	1.4	1.4	1.2
	Moderate	High	53.0	64.8	43.1	65.4	66.4
			1.0	1.2	1.2	1.2	1.0
		Moderate	49.0	63.0	37.8	63.1	63.2
			0.9	1.1	1.1	1.1	0.9
		Low	40.7	56.6	29.2	54.8	57.2
			0.8	0.9	0.9	0.9	0.8
	Low	High	75.1	75.5	56.0	84.1	78.1
			0.8	0.9	0.9	0.9	0.8
		Moderate	71.2	73.7	50.7	81.9	74.9
			0.8	0.9	0.9	0.9	0.7
		Low	62.9	67.3	42.1	73.6	68.9
			0.8	1.0	1.0	1.0	0.8

^a^Covariate age appearing in the model is evaluated at the following value: 45.0.

^b^Covariate age appearing in the model is evaluated at the following value: 45.5.

^c^Covariate age appearing in the model is evaluated at the following value: 45.7.

^d^Covariate age appearing in the model is evaluated at the following value: 45.6.

^e^Covariate age appearing in the model is evaluated at the following value: 45.6.

Keeping age, gender and disability at a constant (the average), increasing from low to moderate and to high PA increased predicted physical health scores from 47.7 to 56.0 to 59.9 (25.6% change from low to high), mental health scores from 60.6 to 67.0 to 68.8 (13.5% change from low to high), energy score from 35.9 to 44.5 to 49.8 (38.7% change from low to high), social function score from 57.8 to 66.1 to 68.4 (18.3% change from low to high), and OQOLS from 58.5 to 64.5 to 67.7 (15.7% change from low to high).

## Discussion

### Summary of findings

This study shows that self-initiated PA, or exercise, is associated with better HRQOL outcomes, regardless of disability level, in a very large, international sample of people diagnosed with MS. Increasing levels of PA were associated with increased levels of energy, social function, physical, mental, and overall QOL as measured by the MSQOL-54 while controlling for age, gender and disability level. Of note, the increases in HRQOL scores, between low to moderate levels of PA, were more pronounced than those between moderate to high levels of PA, indicating that PwMS who are currently participating in low levels of PA may benefit more from increasing their level of PA than those with moderate level increasing to a high level of PA.

An improvement of 5 points on the SF-36, the tool from which the MSQOL-54 is derived, is generally seen as clinically significant [30,31]. The estimated increases of more than 25% on the physical health composite (12.2 points), and estimated increases of around 15% on the mental health composite (8.2 points), social function subscale (10.6 points) and overall QOL subscale (9.2 points) between low and high levels of PA would therefore be clinically significant and of interest to many PwMS. The estimated increase of almost 40% on the energy subscale (13.9 points) is especially noteworthy. Fatigue, related to perceived energy, is a debilitating symptom for most PwMS and is positively affected by PA [32]. The benefits of PA on HRQOL measures are in line with most randomised controlled trials (RCTs) and other studies assessing the association between QOL and PA [15,16,33-37], with one meta-analysis showing the effect of PA on QOL superior to medication[14]. However, at least two RCTs showed no difference in QOL in people with MS after participating in exercise programs [12,13]. Although most studies included only people with mild to moderate disability, a small pilot study including PwMS with high disability showed increases on MSQOL subscales of energy (60%), physical health composite (30.3%), mental health composite (22.4%), and social function subscale (12.6%) (but a decreased score of 2.6% on overall QOL subscale) after a 12 week treadmill training program [38]. The effect of PA on QOL could be mediated through improvements in social participation, fatigue, mood, self-efficacy, perceived stress and/or body functions [5,39-43]. If the type of exercise involves a team or group, it can be expected that PA has a positive effect on perceived social function, whereas being able to complete a training program may improve perceived self-efficacy. However, since QOL is an umbrella term encompassing many of these aspects, it may be difficult to disentangle the relationship between the variables.

Increased level of disability was associated with decreased levels of PA, consistent with the literature [7,8]. Increasing disability and symptoms may prevent PwMS from participating in PA. However, this may be a two-way causal relationship as some evidence is emerging that PA has a disease-modifying effect for PwMS [44], and may have beneficial effects through changes in anti-inflammatory and neurotropic factors in PwMS [45-47] but more research is needed. PwMS generally have lower rates of PA compared to the general population [7,48], even those with mild disability, [49] although evidence is emerging that PA is beneficial for PwMS regardless of level of disability [38,50]. Several RCTs have found PA to favorably affect level of disability [12,51], and a lack of PA may also negatively affect disease progression and characteristics in PwMS [9,10]. The current study design does not allow separating the effects that PA and disability have on each other but future longitudinal data of this cohort may provide further insights.

Due to these expected interactions, regression analyses controlled for disability levels, as disability is expected to affect PA levels as well as the outcome measures. For the subsample of people with relapsing-remitting MS, disease activity was not associated with level of PA and although increased PA was associated with decreased 12 month relapse rate, it was not a significant predictor of relapse rate in the regression analysis when controlling for age, gender and disability level. A recent literature review regarding the effects of PA on disease progression concluded that the evidence from intervention studies was not strong enough to draw definite conclusions but MRI data and self-reported data from PwMS show a possible disease-modifying effect [44]. A longitudinal survey of PwMS found that those with higher baseline activity levels compared to those who had lower levels at baseline showed less functional decline over time suggesting that PA may favorably affect disease progression [43]. Although a survey of PwMS reported that 15% of respondents indicated that aerobic exercise worsened their MS symptoms, conversely 16% reported improvement [52]. There is no other evidence that PA has a deleterious effect on MS [6,53], and a recent study has reported that PA is not related to relapses [54]. Our data also indicated that for PwMS, with low to moderate disability level, increased PA was generally related to a healthier BMI, which is expected to result in better physical health overall [9].

### Limitations

The sample in this study consisted of volunteers from English speaking backgrounds with access to the internet and with the ability to complete this survey online. Most participants were female and between the ages of 38 and 53 years, and thus our population may be less representative of the heterogeneous spectrum of PwMS. It may therefore not be generalizable to all PwMS, despite the number, and variety of backgrounds of participants. All measures were self-reported and it is therefore not possible to assess accuracy or reliability. Due to some incomplete data in the duration items of the IPAQ, a small number of people who indicated to participate in activities once or more per week may have been incorrectly classified in the low level PA group. Although there were 235 severely disabled PwMS who completed PA related items, only 26 and 16 reported moderate and high levels of PA, respectively, which reduced the power to detect significant differences in this group.

IPAQ, the instrument widely used for studying PA in PwMS, has been shown by some to overestimate the intensity and duration of physical activity in the general population [55] and it has been reported that PwMS have difficulties recalling the duration of activities in retrospect as required for the IPAQ [22]. Also, the level of exercise relative to someone’s ability and fitness was not taken into account, rather the IPAQ measures an objective standard of intensity of exercise and this may therefore not accurately measure activity efforts in people with high levels of disability. Further, certain types of PA may be more beneficial than others, and effects may differ between types of MS; this study did not differentiate between these, but future planned research may be able to elucidate this.

## Conclusion

Increased PA was associated with markedly better health related quality of life, including physical and mental health, and especially increased energy, at all levels of disability. Many PwMS report fatigue as a major symptom, and feel they should conserve energy and avoid strenuous activity. Our results add to the growing body of literature suggesting that PA may actually improve perceived energy levels. Clinicians treating PwMS should consider recommending increasing physical activity for PwMS, including those with disability, as it is likely to be associated with improved HRQOL.

## Competing interests

Professor Jelinek receives royalties from his book “Overcoming Multiple Sclerosis: An Evidence-Based Guide to Recovery”.

## Authors’ contributions

CM carried out the statistical analysis and drafted the manuscript. GJ conceived the study. EH contributed to the design and coordinated the study. NP and TW contributed to the study design. DvdM, EH, GJ and TW contributed to the drafting of the manuscript. All authors read and approved the final manuscript.

## Pre-publication history

The pre-publication history for this paper can be accessed here:

http://www.biomedcentral.com/1471-2377/14/143/prepub
